# Interventions in Reducing Caesarean Section in the World: A Systematic Review

**DOI:** 10.21315/mjms2019.26.5.3

**Published:** 2019-11-04

**Authors:** Farideh Moradi, Aidin Aryankhesal, Mohammad Heidari, Ali Soroush, Sara Rahimi Sadr

**Affiliations:** 1 Life Style Modification Research Center, Imam Reza Hospital, Kermanshah University of Medical Sciences, Kermanshah, Iran; 2 Department of Health Services Management, School of Health Management and Information Sciences, Iran University of Medical Sciences, Tehran, Iran; 3 Community-Oriented Nursing Midwifery Research Center, Shahrekord University of Medical Sciences, Shahrekord, Iran

**Keywords:** pregnant women, caesarean section, childbirth

## Abstract

Caesarean section without medical indication imposes many problems to families, personnel and medical equipment causing some side effects to pregnant woman and foetus, compared to natural childbirth. The present study aimed to evaluate the interventions in reducing caesarean section in the world. This study was a systematic review using Embase, PubMed, Scopus, Web of Science, Science Direct, Magiran and SID databases and grey literature. All studies conducted during 2000–2018 were reviewed and finally the studies with inclusion and exclusion criteria were selected. A total of 19 studies were selected among 5,559 studies. The interventions conducted for reducing caesarean section included training the specialists and women by using Six Sigma method, changing the guidelines, reviewing the definition of natural childbirth various stages, encouraging the natural childbirth and expanding painless childbirth. All interventions were divided into educational strategy and managerial strategy. The interventions can be implemented to change the behaviour of physicians and attitude of pregnant women in order to reduce caesarean section. In this regard, the authorities are recommended to make more efforts.

## Introduction

Childbirth is one of the most sensitive and important services of the health system in all societies and caesarean section is considered as one of the main concerns in this regard (1). Caesarean section refers to the removal of the placenta, embryo and membranes by cutting the abdominal wall and uterus (2). Although caesarean section has played a significant role in reducing the mortality and morbidity of high-risk natural childbirth in the last century, its high level is a challenging issue during the recent decades (3).

Caesarean section, without medical indication, creates a lot of troubles on families, medical personnel and medical equipment which can lead to side effects for pregnant women and foetus compared to natural childbirth including the problems related to anaesthesia, post-operative infection, high haemorrhage and thromboembolism (4–6). In addition, caesarean section can have some side effects for infant such as respiratory problems, hospitalisation at the NICU, the possibility of infection, asthma in childhood and reduced breastfeeding (7). Some studies indicated that the reduction of caesarean section affects the maternal and neonatal mortality (8–10).

Further, caesarean section leads to more costs than natural childbirth (11, 12). Douangvichit et al., in their study, at two public hospitals in Latos indicated that the average cost for caesarean section and natural childbirth is 270 and 59 dollars, respectively (13). Based on the World Health Organization (WHO) statistics in different countries, the expected caesarean section should be 10%–15% and it is announced that there is no reason to increase the caesarean section (14). Based on the latest reports from 150 countries in the world, 18.6% of childbirths in the world are performed by caesarean section which is from 6.6% in developed countries and 27.2% in less developed countries (15).

Considering its effects on maternal health and reducing the cost of treatment and the economy of the family and country, most governments adopted some measures to reduce this problem. Such policies and interventions include the changes in the steps of natural childbirth leading to some caesarean section decisions and free natural childbirth (16). Since no study has been conducted on the interventions and policies implemented in different countries to reduce caesarean section, this study aimed to review the policies adopted in this area and evaluate their effectiveness.

## Methods

### Study Design

This systematic review study was conducted according to the Preferred Reporting Items for Systematic Reviews and Meta-Analyses (PRISMA) guidelines (17). The present systematic review study aimed to examine the interventions to reduce caesarean section in the world during 2000–2018 through searching in Embase, PubMed, Scopus, Web of Science, Science Direct, Magiran, SID and grey literature.

### Search Strategy

The key words and search strategy were as follows:

- caesarean or caesarean section or repeat caesarean section or pregnancy or pregnant woman or parturition or birth or labour; and
- intervention or policy or plan or programme or, strategy or strategic planning; and
- decrease or change or impact or health impact assessment.

### Eligibility Criteria

The inclusion criteria for selecting the studies related to the field of research included: i) the studies published during 2000–2018 ii) the studies in English or Persian and iii) the studies referring to at least one aspect of policy interventions to reduce caesarean section. The exclusion criteria included the letter to the editor, commentaries and the articles presented at the conferences.

### Quality Assessment

After extracting the articles from the abovementioned databases, they were evaluated using the descriptive-analytical, Strengthening the Reporting of Observational Studies in Epidemiology (STROBE) checklist and the articles without any good quality of reporting were excluded.

## Results

**Figure 1:**
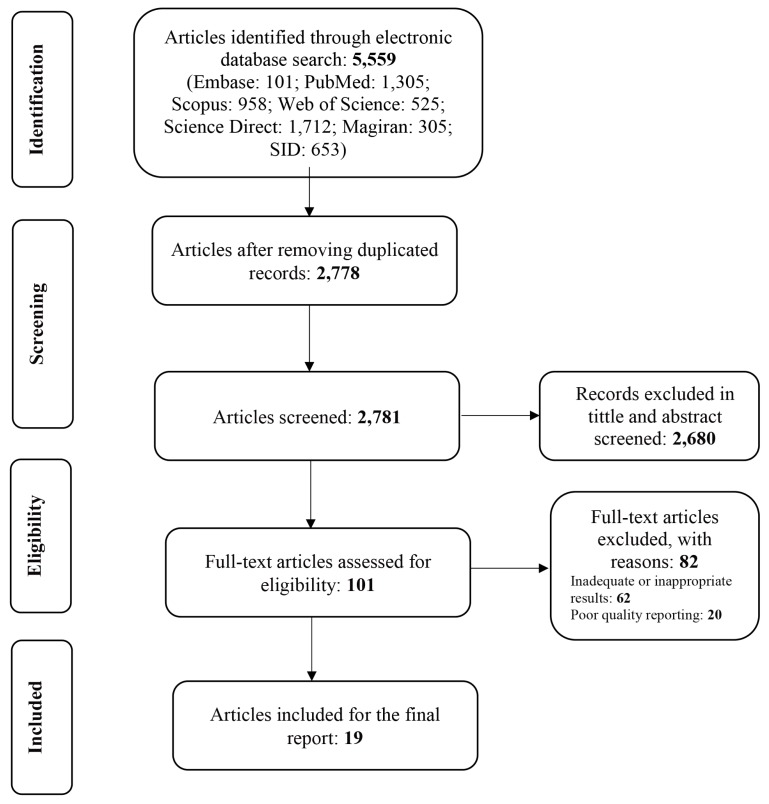
Review selection process and results based on the PRISMA guidelines

A total of 5,559 articles were reviewed, among which 2,778 articles were repetitive. A total of 2,781 articles were screened of which 2,680 articles were eliminated in the review of the title and abstract. Then, the full text of 101 articles was studied among which 82 articles were excluded (62 articles due to inadequate results and 20 articles due to poor quality). Finally, 19 articles were included. The abovementioned items are listed in Figure 1.

### Data Extraction

After reviewing the quality of articles, 19 articles were eventually reviewed by three individuals. The information obtained from the articles were included in Word software and in the table containing the author’s name, year of publication, sample size, analysis method, type of intervention, method of investigation, the most important results and conclusion.

### Summary of Reviewed Articles

A total of 19 articles conducted in different parts of the world during the year 2000–2018 were evaluated after screening in terms of quality and relevance. The processes of reviewing the studies are presented in Figure 1.

Regarding to the target population of the studies, 11 studies were conducted in hospital, 6 studies in health centres and 2 studies in other places. Most studies were conducted after the year 2010 (76%). Asia (67%) allocated the most studies. In terms of research methodology, most of the studies were interventional (67%) and 21% were retrospective studies related to the evaluation programmes, which were already conducted while 12% of the studies were combined. The context of other studies is shown in Table 1.

The strategies obtained from the results of the studies were divided into managerial and educational strategies.[Table t2-03mjms26052019_ra2]

### Educational Strategy

This strategy is a collection of methods and tools for raising the awareness of pregnant women, their families and specialists. Based on the current studies, this strategy has the following sub-categories:

- Training the pregnant women and their families
- Training the specialists
- Applying an experienced person to accompany and train pregnant women in the hospital
- Training the benefits of natural childbirth and side effects of caesarean section to the husbands of pregnant women

### Managerial Strategy

- Performing natural childbirth as free
- Encouraging natural childbirth with the spread of painless childbirth
- Studying and making decision for caesarean section by experienced physicians in the hospital
- Having confidential correspondence with surgeons for conducting caesarean section in the hospital and the surgeon himself
- Using the Six Sigma method
- Changing the guidelines and defining the various stages of childbirth

## Discussion

The results of this study indicated that education is effective in reducing caesarean section. The results of this study are consistent with studies of Spinelli et al. (18) and Ferguson et al. (19) but inconsistent with the studies of Artieta-Pinedo et al. (20) and, Bostani and Rafat (21).

It seems that the differences in the studied population such as the level of education, attitude of people and the readiness of people to attend educational classes are not affected by the difference between the results obtained from these studies. Other factors affecting the outcomes of education includes the skills and experiences of the trainer, the extent he addressed all the aspects of the subject and whether he was able to transfer it to the learners. In addition, the number of learners and their interest in learning, the time and number of hours spent in the classroom are influential in this regard.

The findings of this study indicated that training the husbands is effective in reducing caesarean section. Noghaee and Hadizadeh indicated that implementing the educational programme and raising the awareness of men can be effective in social protection of women (22).

Considering that men are one of the important pillars of reproductive health services, their role as the closest person to their spouse was confirmed in supporting the women and running family planning programmes. Thus, it is suggested that men should be trained and justified to transfer the learned knowledge to their spouses, which results in increasing women’s awareness and reducing caesarean section.

Sharghi et al. indicated a significant relationship between the opinion of the spouse and the desire to choose caesarean section (23). In another study, Faraji Darkhaneh and Farjad Bastani indicated that 64% of women considered the role of the spouse in choosing the method of childbirth (24).

The results of this study indicated that using a midwife is effective in reducing caesarean section. As a result, the presence of a midwife after the childbirth, facilitates the pregnant women readiness for infants and postpartum affairs. Furthermore, the results of Kozhimannil et al.’s study indicated that the presence of a midwife is effective in reducing caesarean section (25).

Considering the role of midwife in the care of pregnant women before, during and after childbirth, as well as preventing the complications and risks of pregnancy by this group of people, it is recommended to pay more attention to this issue and provide special conditions and facilities for using pregnant women including the easy and inexpensive access.

The results of this study indicated that the evaluation and indication of caesarean section as well as using the feedback to specialists had a slight effect on the rate of caesarean section. The results of Khunpradit et al.’s review study (26) are in line with the results of this study. The results of Tavarez et al.’s study indicated that the feedback of performance to pediatric emergency physicians over a three-month period is ineffective in the management of patients with diarrhea and vomiting (27). In another study, Tu et al. confirmed that the announcement of hospital indicators is ineffective in improving the indices (28). The results of these studies were inconsistent with those in the present study.

Based on the results of a meta-analysis study, feedback and evaluation were effective at a rate of 13% in reducing the caesarean section (29). In addition, the results of some studies are not in line with those in the present study (30–33).

It seems that the underlying characteristics of the environment where feedback is performed including the amount of personnel’s attention and belief in feedback is not ineffective in the obtained results. Sargeant et al. indicated that the reaction and perception of physicians to multi-stage feedbacks can affect their performance (34). However, the characteristics of service providers, the way of providing feedback and the extent of real feedback to personnel may not be ineffective in their feedback.

Physician-patient relationship culture in Iran indicates that medical team can play an important role in controlling or stimulating caesarean statistics. It is suggested that physicians should always be given the necessary feedback in this regard. Obviously, this control has a moral value physically and economically. The findings of this study stated the level of tariffs on caesarean section reduction. The results of the study by Fabri and Murta confirmed this finding (35).

Interventions in the developed countries (change in guidelines) were more systematic in this study and involved a wide range of women, leading to the better process of natural childbirth and caesarean section reduction. However, the interventions in developing countries including Iran had a personal dimension including only a few people who are learners. The lack of transparency in the research method in a limited number of studies was one of the limitations encountered by the researchers in this study. It is suggested that future studies should include the interventions, which are more systematic and functional.

## Conclusion

Finally, the researcher believed that modifying the referral system for childbirth is considered as the most important method for reducing the caesarean section. All the patients should first be examined by midwife and referred to a specialist in case of necessity and the need for the indications of caesarean section. Further, training the pregnant women and their husbands, performing natural childbirth as free, studying and making decision for caesarean section by experienced physicians in the hospital, encouraging natural childbirth with the spread of painless childbirth are considered as effective.

## Figures and Tables

**Table 1:** Countries that studied

Country	Number of studies
Iran	10 studies
China	2 studies
US	2 studies
France	1 study
India	1 study
Pakistan	1 study
Taiwan	1 study
Argentina, Brazil, Cuba, Guatemala, Mexico	1 study

**Table 2 t2-03mjms26052019_ra2:** Summary of data extraction of the selected articles to investigate investigating the interventions in reducing caesarean section in the world (2000–2018)

Authors & Year	setting	Intervention	Sample size	Statistical analysis	Methods	Result	Conclusion
Asadi et al. (2014) (36)	Iran	Training by question and answer method, video presentation and lecture for 60 min in the form of group meetings with at least dive pregnant women who intended to perform caesarean section, holding a session with the presence of physicians and influential people including the learners’ husbands, distribution of a book entitled “Reproductive Health” to remind the contents to the learners	90	Using statistical software (SPSS version 16) and chi-square, Mann-Whitney, Wilcoxon, *t*-test and paired *t*-tests	The study type was semi-experimental, prospective and case-control. The inclusion criteria included in the third trimester of gestation with a gestational age of 28–24 weeks, without a history of childbirth, with a high tendency to caesarean section and without a medical avoidance for natural childbirth-special training in the case group and normal training in the control group, the sampling was conducted in three areas of down, centre and top of the city and selecting 15 people in each area	After the intervention, seven (15.6%) subjects in the case group had the intent to choose natural childbirth but it did not happen in the control group	The theory of reasoned action can be effective in creating and increasing the intent of pregnant women to choose natural childbirth
Azh and Yoonesian (2014) (37)	Iran	Training the case group including the use of slides and video tapes about the anatomy and physiology of pregnancy, preparation for natural childbirth and caesarean section, method of reducing pain, formation of a group discussion about the senses and attitudes of the patient in connection with childbirth, invitation of the husbands of the pregnant women, training them about postpartum problems and appropriate solutions to them, distribution of educational pamphlets on postpartum education, three training sessions for two hours: The inclusion criteria included: i) the last three months of pregnancy, ii). first or second pregnancy, iii). no history of illness and depression, iv). not having a child, v). age between 17 and 34 years, vi). having at least the middle school degree, vii). confirmation of the health of the foetus in ultrasound examination	410	Chi-square test	The study type was case-control and interventional, the case group included 187 subjects and control group included 223 subjects. Providing special training to the case group and regular training to the control group	The rate of caesarean section was 41.41% in the case group and 74.8% in the control group and the overall incidence was 59.8%. The caesarean section was 60.5% in the control group and 16.7% in the case group, and this difference was statistically significant (*P* ≤ 0.001)	Placing a coherent curriculum along with other social support methods can be effective in reducing caesarean section
Taheri et al. (2014) (38)	Iran	Training the pregnant women in three sessions of 60 min–90 min. Session 1: group discussion of pregnant women with one another on the cause of fear of delivery. Session 2: presentation of the successful natural pregnancy experience by pregnant women and education on the anxiety of pregnant mothers on the embryo, caesarean section, and Kegel exercise. Session 3: repetition of the training	130	Paired and independent *t*-test, chi-square and variance analysis.	The study type was quasi-experimental. The population included pregnant women	After the intervention, the mean score of childbirth in the intervention group decreased and the mean score of waiting for childbirth and the efficiency of childbirth increased. 71.4% of the pregnant women of the intervention group and 53.8% of the pregnant women of the control group were given natural childbirth	Providing the necessary training during pregnancy was effective in encouraging pregnant women to have natural childbirth
Bogg et al. (2016) (39)	India	Paying some money to maternity specialist to encourage childbirth. In the state of Madhaya, the fee paid for caesarean rates was higher than natural childbirth and the fee was ascendant while in the state of Gujarat, a fixed fee was paid to specialists based on the childbirth type	805,000	Descriptive statistics analysis.	The study compared two public and private funding programs in two Indian states which were conducted for increasing the number of childbirth	In the state of Madhaya, the rate of caesarean section increased from 26.6% in 2007 to 40.7% in 2010, while the rate for caesarean section was 4.9% before the program was implemented. In Gujarat, the rate of caesarean section was reduced 4.3% than in 2004 which was 0.8%	Findings indicated that financial incentives affect the selection of childbirth type
Chai et al. (2017) (40)	China	Monitoring and final decision making for senior resident caesarean, diabetes screening and prevention education for preventing diabetes and fetal enlargement and reducing caesarean section, training on neonatal resuscitation and natural childbirth, postpartum haemorrhage, childbirth using forceps by midwives as monthly	1,016	Using Minitab software X2 test to compare classification information.	The study type was interventional-sampling and extracting information from archival files, determining the causes of caesarean section using fish bone charts as one of the Six sigma stages, determining intervention based on caesarean section	The defect rate declined from 41.83% to 32% and the Six Sigma score increased from 1.706 to 1.967 after improvement measures (*P* < 0.01)	Six Sigma is an effective approach to reduce caesarean section
Ganjee and Khosravi (2008) (41)	Iran	Training the pregnant women on the benefits of natural childbirth and complications of caesarean section by the health care team including midwives, health clinic physicians, and non-sanitary staff	171	Paired *t*-test and McNemar test	The study was field type and the research population included the pregnant women having no previous history of caesarean section and less than three times of pregnancy who were randomly selected and given the necessary training	In this study, 70% of pregnant women were satisfied with the provided training. The frequency of caesarean section decreased from 63% before the implementation to 52% after intervention (*P* < 0.05)	In some cases of health problems such as caesarean section which is rooted in the culture and beliefs of the people, the mobilisation of groups of people in solving the problem, along with the promotion of related health services, can have beneficial effects
Ghaffari et al. (2011) (42)	Iran	Educational programme was understood during six sessions according to the health belief model, including awareness, perceived sensitivity, perceived severity, perceived barriers, perceived benefits, and self-efficacy. The information was provided by film and lecture	100	Using SPSS 14 and independent-*t* and *t-*paired tests, chi-square, and Mann–Whitney test	The study type was semi-experimental, the research population included pregnant women delivering their first child who were at the 20–30 weeks of gestation. The instrument of this study was a questionnaire based on the structure of the health belief model, intervention in the case group and completion of the questionnaire 4–8 weeks after intervention in both groups	There was a significant difference in the mean score of awareness (*P* ≤ 0.001), Perceived sensitivity (*P* ≤ 0.001), Perceived severity (*P* ≤ 0.001), Perceived benefits ( *P* ≤ 0.001) and Perceived self-efficacy ( *P* = 0.02). There was no significant correlation between women in the case group than in the control group after intervention. In the case of perceived barrier structures (*P* = 0.09), there was no significant difference in the choice of childbirth type (*P* = 0.73) and performance (*P* = 0. 24)	Educational programme based on health belief model is effective in increasing the knowledge and attitudes of pregnant women in childbirth
Karami et al. (2017) (43)	Iran	Natural childbirth as free, the encouragement of natural childbirth with the spread of painless childbirth, the reconstruction of childbirth blocks by creating special spaces for the pleasantness of the natural childbirth environment	Fifteen hospitals affiliated to Kermanshah University of Medical Sciences	Discontinued time series.	The study type was retrospective and quasi-experimental. The statistical population included the hospitals affiliated to Kermanshah UMS and the number of caesarean section was evaluated 25 months before and 28 months after the intervention	Caesarean section decreased to 0.11 in the first month after the interventions but it increased monthly to 0.17 (*P* ≤ 001)	Although the rate of caesarean section declined in the first month after the implementation of the health plan, its rate increased during the study. This suggests that natural childbirth in Iran has not been increased as one of the goals set before the implementation of the plan
Mawson (2004) (44)	Argentina, Brazil, Cuba, Guatemala, Mexico	The indications were examined using existing evidence from physicians being higher or at the same level in terms of the clinical experience and quality and the final decision was made by the physicians by drawing the flowchart	36 hospitals		This study was a randomised, controlled cluster clinical trial study. The population included the hospitals in Latin America with caesarean section rate as high as 15% per 1000 cases per year	In the hospitals where intervention was performed, the rate of caesarean section decreased (1.9% to 1% *P* = 0.044). 87% of physicians believed that this was an effective strategy in public hospitals and 41% believed that they were effective in private hospitals	Intervention of using the second physician’s opinion was effective in reducing the number of caesarean section
McGrath and Kennell (2008) (45)	US	Intervention involved the use of a trained and experienced person to accompany and support pregnant women after admission to the hospital. This support is provided in the physical proximity, eye contact and communication, training, assurance and insurance, and encouragement of pregnant women and their husbands	420	Descriptive analysis, analytical analysis for comparing intervention and control groups - Chi-square test for discrete variables analysis.	This study was a controlled clinical trial. The research population included the pregnant women during their third trimester. A total of 420 pregnant women were included in the study of whom 196 women were in the control group and 224 women were randomly assigned to the intervention group	In the intervention group, the number of caesarean sections decreased compared to the control group (13.4% versus 25%, *P* = 0.002). In the women with induction childbirth, the supported group had lower than caesarean section (12.5% versus 58.8%, *P* = 0.007) and less number of pregnant women in the intervention group received epidural anesthesia than the control group (64.7% versus 76% , *P* = 0.002)	For the middle class women working with the support of their husband, the continuous attendance of a midwife in the hospital reduces the likelihood of caesarean section and the need for epidural anesthesia
Naiden and Deshpande (2001) (46)	US	Using oxytocin and its injection under the supervision of a physician, examination of the condition and fetus of the fetus before the injection of oxytocin, implementation of the protocol for high-risk childbirth by experienced midwives, use of intrauterine catheter by doctors if the dose of oxytocin is more than 20		The statistics were divided into ten periods of one year, using the *X*^2^ test with a factor of 2 at 10	The study type was retrospective evaluating the rate of caesarean statistics over ten years (1989 to 1998) and the factors reducing this rate	The rate of caesarean section ranged from 16.59% to 10.92% and the initial caesarean was from 9.22 to 7.11 while the repeated caesarean section ranged from 7.37% to 3.81%. The rate of natural childbirth after caesarean section increased from 35.6 to 54.5 (*P* < 0.001). The use of oxytocin increased from 38.8 to 63.4 (*P* < 0.001)	The found that our working plan for management of labor and delivery yielded and maintained a successful decline in the cesarean delivery rates without any negative effect on neonatal or maternal mortality rates
Peng et al. (2016) (47)	Taiwan	Evaluating the causes and the need for caesarean section by a team of 8 experts and presenting results at monthly conferences	2,189	Wilcoxon test, logistic regression	This study was a retrospective study. 3,781 individuals who were given treatment from January 2008 to January 2011 were studied Mothers who had given birth from January 2008 to January 2009 were part of the pre-audit group (1,592) while those who had given birth after August 2009 were part of the post-audit group. Then, the rate of caesarean section was evaluated in two groups	Caesarean section due to Dystocia 9.6 versus 2.6 (*P* ≤ 0.001) was significantly less in the clinical audit group than in the pre-audit group. However, there was no significant difference in the vaginal delivery rate among the groups; of 195 cases, 16 audited caesarean sections did not require caesarean section. In non-delivered women (2148 cases), multivariate analysis indicated that clinical examination (OR = 0.78), maternal (OR = 1.10), gestational age at delivery (OR = 0.80), and embryo birth weight (OR = 0.005) were independent of caesarean section by default *P* ≤ 0.005)	Clinical audit is an effective strategy for reducing caesarean delivery
Piroozi et al. (2016) (48)	Iran	Natural childbirth for free, the encouragement of natural childbirth with the spread of painless labor, the reconstruction of delivery blocks by creating special spaces for the pleasurable childbirth environment, rewarding the delivery team	1,155	Frequency indices, percentage, mean and chi-square test	The type of retrospective and longitudinal study was the study population of all hospitals in Kurdistan province having a maternity ward, research tool is check list	14.02% of the rate of caesarean section decreased after the implementation of the health system reform plan	After implementing the health care reform plan, the rate of caesarean section 0.10 decreased in comparison to before the implementation
Khani (2004) (49)	Iran	Confidential correspondence with surgeons regarding the rate of caesarean section performed in the hospital and the rate of caesarean section performed by each surgeon		Descriptive statistics (frequency, mean, standard deviation) and inferential (mean comparisons and *X*^2^)	Type of clinical trial, the research population including two selected hospitals in Mazandaran province (one hospital in case of a control hospital), 10-month intervention, comparison of data before and after intervention in each hospital	During the study, 2,171 childbirths were conducted in the case hospital and 980 childbirths were conducted in Shahed Hospital and 44.8% of childbirths were in the case group and 46.6% of the childbirths were in the control group by caesarean section. The test indicated that the intervention did not affect the rate of caesarean section, but the rate of caesarean section decreased in the case and control groups after intervention (P < 0.001)	Supervision and control of the authorities is required for the rate of caesarean section, and it is better for other organisations to co-operate with the treatment department to reduce caesarean delivery
Safari Moradabadi et al. (2014) (50)	Iran	Training based on pre-test analysis on the maternal fear from natural childbirth, caesarean section disadvantages and natural childbirth advantages, in several 50–60 min training sessions (number of sessions depending on the characteristics of the target group), lecture, group discussion, question and answer, PowerPoint software for the intervention group. At the final session, the film on two types of natural childbirth and caesarean section were shown to the participants	70 (35 subjects in the intervention group and 35 subjects in the control group)	Descriptive statistics, inferential statistics (independent *t*-test and paired *t*-test)	The study type was interventional with two intervention and control groups. The study population included all pregnant women having their first child (with caesarean section intention) simple random sampling, inclusion criteria in both groups, the pregnant women having their first child wanted caesarean section during 25–30 weeks of gestation, no clear barriers to natural childbirth, exclusion criteria in both intervention and control groups, including the absence of pregnant women during training sessions (absence more than two sessions), the unavailability of pregnant women, incomplete questionnaire, and termination of pregnancy for any reason, data collection is a structured questionnaire based on the research objectives and the use of scientific sources	A significant difference was observed between the awareness scores (*P* < 0.001) and self-efficacy ( *P* < 0.001) after educational intervention in the two groups. After the educational intervention in the intervention group, 16 (4.45%) women selected natural childbirth as the preferred method. After telephone follow ups, 11 (42.31%) women selected natural childbirth	The design and implementation of systematic training programs by health workers has a significant role in encouraging pregnant mothers to undergo natural childbirth and reduce caesarean section
Sharifirad et al. (2013) (51)	Iran	Dividing the husbands of the case group into three groups of 13–15 subjects, duration of training for 90 minutes, educational content of the mechanism of natural childbirth and caesarean section, their disadvantages and advantages, training method (lecture and Q & A sessions)	88 pregnant women	independent *t*-test, paired *t*-test, chi-square test, ANOVA	The study type was experimental and the research population included the pregnant women at 28–32 weeks and 4 weeks after training, the knowledge and awareness of the studied group (case and control group) was evaluated	Caesarean section in the case group was much less than the control group (29.5% versus 50%)	Training the husband of pregnant women can be effective in the knowledge and positive attitude of spouses for natural childbirth and reduction of selective caesarean section
Sheikh et al. (2008) (52)	Pakistan	Evaluation of caesarean section and presentation of results in form of instructions to relevant departments. Evaluation checklist including maternal age, delivery status, delivery period [beginning of delivery, emergency level, necessity and need for postoperative monitoring, postoperative complications, etc.]	No sampling was conducted.	No statistical test was performed	This study was a comprehensive evaluation of all primary caesarean sections from January 1 to March 31 during 2003–2004 and the results of the evaluation were presented in the form of a guideline. Then, the rate of caesarean section and maternal and fetal status were checked before and after the implementation of the guideline	The rate of caesarean section decreased from 16% to 12%. The exercise of examining the fetal cord blood and maintenance of parameters improved and there was no maternal and perinatal consequence	Implementing the standard childbirth management can reduce primary caesarean sections without hurting the maternal and fetal safety
Thuillier et al. (16)	France	Changing the guidelines or changing the definition of the various stages of natural childbirth	The participants were 3,283 people before intervention and 3,068 people were after intervention	Statistical analysis using R studio version 0.99.896 (CRAN) software, *X*^2^ tests, Fisher test, odds ratio and independent T	The study type was before-and-after cohort. The study population included the pregnant women having their first child with a gestational age of more than 37 weeks. The study place was one of the university affiliated hospitals	The rate of caesarean section decreased significantly from 9.4% to 6.9%. In the period after the change in the guidelines (*P* < 0.01), the rate of caesarean section in the first stage was less than half, 1.8 to 0.09. It decreased in the second phase but was not significant between periods. In addition, the duration of the decision to perform caesarean section with regard to the cervical position was significantly longer in the first childbirth	Changing the protocol resulted in a decrease in caesarean section without any complication in the infants
Yu et al. (2017) (53)	China	Policy interventions including the Implementation of evaluation programs such as the indicators of control and quality management for comprehensive hospitals, and ultimately the impact of these policies on encouraging mothers to carry out natural childbirth by the health care team. Organisational interventions include education for mothers and their families by physicians and nutritionists once or twice a week, training through billboards, caesarean section by specialist teams, participation of midwives in educational classes annually, use of painless childbirth in the hospital	312,131	Chi-square test and predicted model	A before-and-after study that was conducted during 2006–2014 in three clinical clinics in China	After applying organisational interventions, the rate of caesarean section decreased by 12%. The average annual growth of this index decreased from 20.11 to −4.30. After policy interventions, the overall caesarean section rate and annual probability decreased	Organisational and policy interventions can reduce the amount of proposed caesarean section and this index should be considered as one of the indicators for assessing the hospitals
